# Multi-Stakeholder Primary Health Care for Migrant Populations in Thailand’s Border Regions: A Qualitative Study of Barriers and Opportunities

**DOI:** 10.1007/s10903-025-01845-0

**Published:** 2026-01-04

**Authors:** Kitti Sranacharoenpong, Bang-On Thepthien, Pyae Phyo Kyaw, Mathuros Tipayamongkholgul

**Affiliations:** 1 Asean Institute for Health Development, Mahidol University, Nakhon Pathom, Thailand; 2 Research Institute for Languages and Cultures, Mahidol University, Nakhon Pathom, Thailand

**Keywords:** Primary health care, Universal health coverage, Migrant health, Cross-border health governance, Southeast asia, Greater Mekong-Subregion

## Abstract

Primary health care (PHC) in Thailand’s border regions remains challenged by the needs of mobile and marginalized populations, despite the country’s progressive Universal Health Coverage (UHC). This qualitative study explores the dynamics of cross-border PHC systems along Thailand’s borders with Myanmar, Lao PDR, and Cambodia through a people-centered lens. We conducted 101 semi-structured interviews with diverse stakeholders across six border provinces. Findings reveal that migrants and refugees face complex barriers, including legal exclusion, geographic isolation, and fear of deportation, leading to reliance on self-medication and delayed care. Health priorities consistently included communicable diseases, maternal and child health, and occupational risks, yet the context varied significantly by border. The Myanmar border was characterized by Non-Government Organizations (NGOs)-dependent, fragmented services for undocumented migrants and refugees. In contrast, the Lao PDR border demonstrated more stable, seasonal migration and smoother bilateral health cooperation. The Cambodia border was defined by labor migration and inconsistent employer-based health arrangements. Multi-stakeholder collaboration—particularly through migrant health volunteers and NGOs—was identified as both feasible and essential for service delivery, but remains ad hoc and donor-dependent. Sustainable solutions require formalizing the roles of community-based actors, creating institutionalized coordination platforms, and developing tailored, context-specific strategies that address the fundamental social determinants of health. Achieving health equity in these borderlands necessitates inclusive policies that bridge the gap between national UHC ambitions and the realities of cross-border mobility.

## Introduction

The borderlands of the Greater Mekong Region (GMR)—where Thailand meets Myanmar, Lao PDR, and Cambodia—are dynamic spaces shaped by mobility, cultural diversity, and systemic inequities [1]. Migrant workers, refugees, ethnic and minorities often face profound barriers to healthcare due to legal restrictions, poverty, and geographical isolation. These populations remain at heightened risk from communicable diseases, maternal and child health problems, and environmental threats [2–4].

Globally, primary health care (PHC) is the cornerstone of equitable health systems. In border regions, its role is especially critical in reaching underserved and mobile populations. Thailand has distinguished itself through universal health coverage (UHC) anchored in PHC [5, 6], providing widespread access and reducing out-of-pocket spending [7–9]. Yet, disparities persist across borders where health systems remain fragile, donor-dependent, and fragmented, prompting many to cross into Thailand for care. Additionally, the border health service system is required culture-sensitive responsiveness to patients to enhance accessibility to care and improve health and wellbeing of people along the border [1–4].

Despite some promising initiatives, such as mobile vaccination campaigns and migrant health volunteers, cross-border health governance remains ad hoc and underdeveloped. Weak referral systems, limited data sharing, language barriers, and funding gaps undermine coordinated responses and perpetuate inequities [10–14]. This lack of sustained collaboration highlights an urgent need for approaches that are inclusive, adaptive, and people-centered.

This study adopts a people-centered health systems (PCHS) lens and intercultural adaptation to explore PHC along Thailand’s borders with Myanmar, Lao PDR, and Cambodia. Through qualitative inquiry, it seeks to (1) assess the strengths and weaknesses of PHC in border contexts; (2) examine the feasibility of multi-stakeholder engagement involving government, NGOs, and communities; and (3) analyzes the social determinants- migration, poverty, cultural diversity, and environmental risks—that shape access to health services. By situating local experiences within broader systemic challenges, the study contributes to ongoing debates on cross-border health governance and strategies for inclusive PHC strengthening.

## Methods

### Study Design

This qualitative study was guided by a constructivist paradigm, acknowledging that understanding the complexities of cross-border healthcare requires exploring the multiple, socially constructed realities of diverse stakeholders. We employed an interpretive phenomenological approach to delve deeply into the lived experiences of stakeholders of primary health care services in Thailand’s border regions.

We collected data through semi-structured, in-depth interviews with key informants. This method was chosen to gather rich, detailed narratives and allow for probing questions to clarify and explore emerging themes. Thematic analysis was used to identify patterns across the dataset, and triangulation of data sources was employed to ensure the validity and reliability of the findings. The main interviewer is one of the authors who was trained in qualitative research during her doctoral program and has extensive experience in this field. This report sequence was aligned with the COREQ (Consolidated criteria for Reporting Qualitative research) [15].

### Study Setting and Site Selection

This study was conducted in border provinces of Thailand representing diverse rural and semi-rural contexts along three international borders. The rurality was defined according to the Thai administrative and demographic framework, consistent with international definitions emphasizing low population density, limited access to specialized health services, and dependence on agriculture or small-scale industries [16, 17].

The six border provinces of Thailand, selected through purposive sampling to capture the diverse geographical, socio-economic, and migration contexts along the borders with Myanmar, the Lao People’s Democratic Republic (Lao PDR), and Cambodia. Two provinces were selected on each side of the border to ensure variation in health service delivery, population mobility, and cross-border dynamics.

**Fig. 1:**
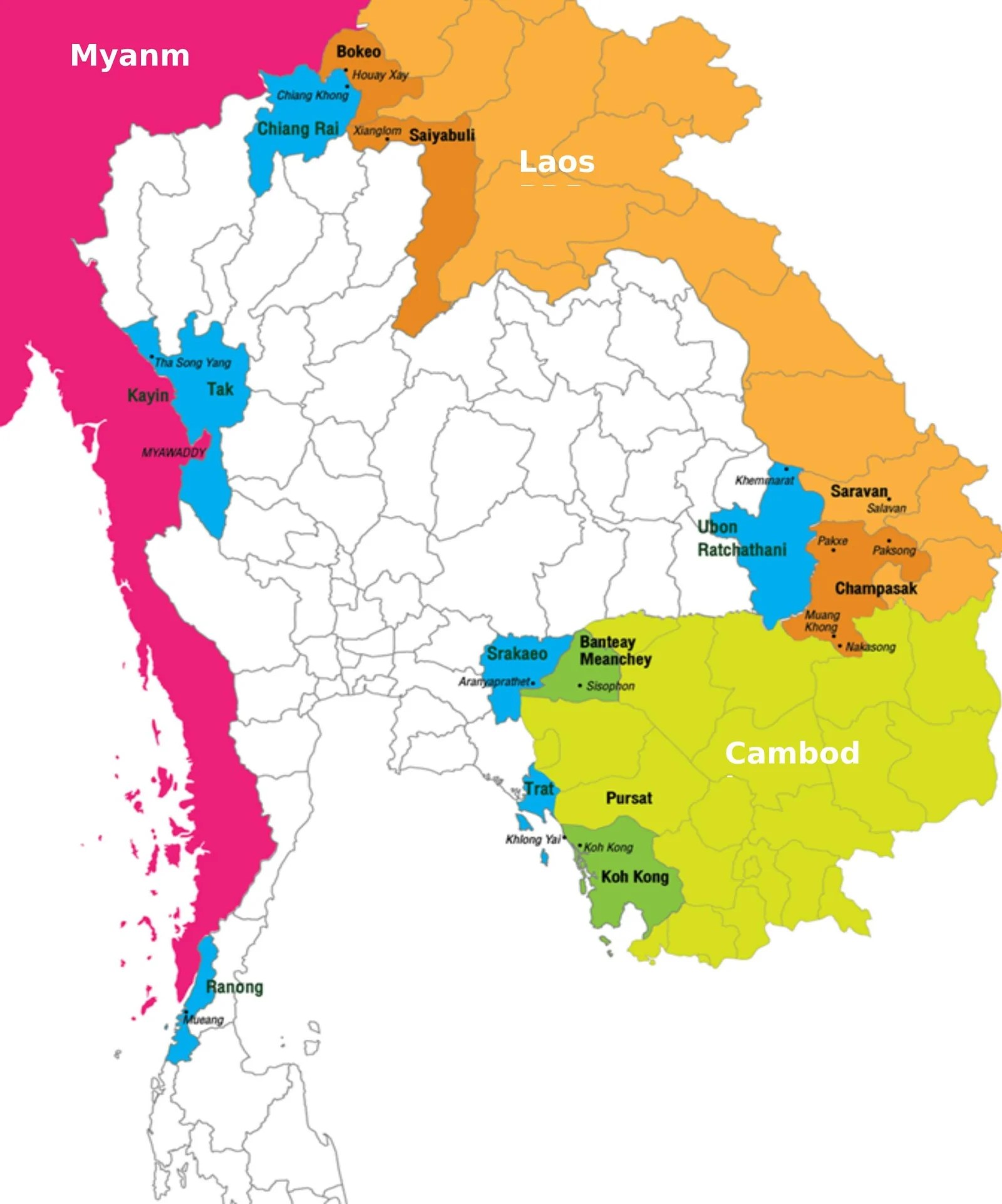
A map of the six study provinces

Thailand–Myanmar Border: Tak Province (particularly Tha Song Yang District), characterized by mountainous terrain, dispersed settlements, and limited transportation infrastructure, and serving as a key hub for migrant workers and refugees [3]. Ranong Province (Muang District) represents a rural coastal province with a high proportion of migrant laborers engaged in fisheries and related industries. Thailand–Lao PDR Border: Chiang Rai Province (Chiang Khong District), a predominantly rural area with multiple border checkpoints for trade and migration, and Ubon Ratchathani Province (Khemmarat District), which represents agricultural and seasonal migration communities with low urbanization levels. Thailand–Cambodia Border: Sa Kaeo Province (Aranyaprathet District), a major land-crossing point for trade and labor migration with a largely rural population [17], and Trat Province (Khlong Yai District), featuring small rural communities and both formal and informal border crossings used by local traders and migrants [17] (Fig. 1).

### Participant Selection and Recruitment

A total of 101 key informants were persons in charge of or engaged in primary healthcare (PHC) delivery from public health organizations, health facilities, local administrative organizations, and civil society, representing the full spectrum of stakeholders involved in PHC delivery and cross-border health initiatives. Key informants from six provinces, across all administrative levels from province, district, and village, were purposively recruited. Initially, a public health staff member responsible for PHC delivery in the study areas was identified, and information provided by this key informant was subsequently used to recruit additional key informants.

### Data Collection

We commenced data collection process in June 2025 and completed it in July 2025. A semi-structured interview guide was developed based on the study objectives and a review of relevant literature. The guide focused on three core domains:

1) Health System Assessment: Experiences with service delivery, perceived strengths and weaknesses of existing PHC systems, and identified gaps for vulnerable populations.

2) Multi-stakeholder Engagement: Past and current experiences with collaboration, perceived barriers and facilitators to partnership, and feasibility of sustained engagement.

3) Social Determinants of Health: Perceptions of how migration, poverty, language, culture, and legal status influence health access and outcomes.

We conducted the in-depth interview at a location chosen by the key informant (e.g., the key informant’s office, a community center) to ensure comfort and confidentiality. Before each interview, written informed consent was obtained. Interviews lasted between 15 and 45 min, were conducted in Thai or local dialect with the aid of a trained interpreter as needed, and were audio-recorded with permission. Field notes were taken to capture contextual observations.

### Data Analysis

Audio recordings were transcribed verbatim and, where necessary, translated to Thai by trained interpreter. The data were analyzed using reflexive thematic analysis following the six-phase approach by Braun and Clarke [18]:

1. Familiarization: Researchers immersed themselves in the data by reading and re-reading transcripts.
2. Generating Initial Codes: Systematic coding was applied to the entire dataset using NVivo 12 software.
3. Searching for Themes: Codes were collated into potential themes.
4. Reviewing Themes: Themes were checked against the coded data and the entire dataset for consistency.
5. Defining and Naming Themes: The essence of each theme was refined and clearly defined.
6. Producing the Report: Vivid, compelling extracts were selected to illustrate the final analysis.

To enhance trustworthiness, triangulation was achieved by comparing perspectives across different stakeholder groups and study sites. Peer debriefing sessions were held among the research team to challenge interpretations and strengthen the analysis [18].

### Ethical Considerations

The study protocol was reviewed and approved by the Mahidol University Human Research Ethics Committee (CAO No. MU_CIRB 2025/141.3004). The study was conducted in accordance with the Declaration of Helsinki. All participants provided written informed consent. They were informed of their right to withdraw at any time without penalty. All data were anonymized during transcription and analysis to protect participant confidentiality. Digital files were stored on a password-protected server.

## Results

A total of 101 key informants were purposively recruited to represent the full spectrum of stakeholders involved in PHC delivery and cross-border health initiatives. Recruitment continued until data saturation was reached, where new interviews no longer yielded novel themes or insights. Of 101 key informants, sixty were from government organizations, 36 from private organizations and civil society, and five from international organizations. We recruited key informants from six provinces, with a range of 14 to 20 persons per province. Of 101, 54.9% are male, and 2 of 3 are aged 40 and above, and hold working experience in the current position for at least 2 years.

This study identified six key themes reflecting the opportunities and challenges of primary health care (PHC) in Thailand’s border regions with Myanmar, Lao PDR, and Cambodia.

### Theme 1: Complex Health Challenges

Stakeholders consistently emphasized that border populations remain among the most underserved in Thailand. Migrants, and refugees were described as “falling through the cracks” of the health system due to a lack of documentation, fear of deportation, and unaffordable out-of-pocket costs. Many rely on self-medication or informal providers, delaying treatment until conditions become critical. A district health officer at Thai-Cambodian border explained, *“Undocumented migrants are reluctant to visit health facilities unless it is an emergency*,* as they worry about being reported to authorities.”*

Geographical isolation and shrinking budgets further compounded access difficulties. A village health volunteer at Thai- Cambodia border noted, *“Some families live hours from the nearest clinic. Without transport*,* they wait until illnesses become severe.”* Participants linked these barriers to higher rates of communicable diseases, maternal complications, and preventable deaths.

### Theme 2: Regional Differences

Marked differences emerged across border regions, and detailed information is presented in Table 1.

**Table 1:** Comparison of Population, Economic, and health systems for Thailand, Lao PDR, Cambodia, and Myanmar

Data	Thailand	Lao PDR	Cambodia	Myanmar
Population	~ 66 million, aging population	~ 7.5 million, young population	~ 17 million, mostly young	~ 55 million, mixed age structure
Birth rate (/1,000)	Low (~ 9)	High (~ 22)	High (~ 20)	Moderate (~ 18)
Population Structure (Aging, % ≥65 years)	High (~ 13%) (Ageing Society)	Low (~ 5%)	Low (~ 6%)	Low (~ 5%)
GDP per Capita (USD)	~$7,300	~$2,600	~$2,000	~$1,300
Poverty Rate (%)	Low (~ 7%)	High (~ 18%)	Moderate (~ 17%)	High (~ 25%)
Health Expenditure (% of GDP)	~ 4%	~ 2.8%	~ 2.5%	~ 1.8%
Life Expectancy	~ 78 years	~ 68 years	~ 70 years	~ 67 years
Under-5 Mortality Rate (per 1,000 live births)	Low (~ 8)	High (~ 45)	High (~ 29)	High (~ 48)
Health Coverage	Universal Coverage Scheme (UCS) covering ~ 98% of the population	Expanding PHC; limited rural coverage	Relies on Health Equity Funds (HEFs) and NGO support	Severely underfunded; dependent on humanitarian aid
PHC System Strength	Strong PHC network; focus on NCDs and elderly care	National Health Reform Strategy to expand rural services	Progress in maternal/child health, but high rural disparities	Weak PHC, critical workforce shortages, poor infrastructure
Key Barriers	NCD management, elderly care	Geographic access, workforce gaps	Out-of-pocket expenses, fragmented rural health system	Political instability, conflict, and lack of financing
Reliance on NGOs	Limited (primarily for migrants/refugees)	Moderate (especially in remote areas)	High in rural areas and for vulnerable groups	Very high; NGOs play a critical role in basic service delivery

Myanmar border: Services depend heavily on NGOs in refugee camps and informal settlements. A local health officer at Tha Song Yang (or Myanmar-Thailand) border observed, *“In the camps*,* basic health services are run mostly by NGOs. Our hospital supports emergencies*,* but many residents have no formal coverage.”*

Lao PDR border: Migration is seasonal and family-based, with more stable cooperation and cross-border vaccination initiatives. *“Families cross during harvest but return regularly*,* so it is easier to follow up with care*,*”* explained a public health nurse.

Cambodia border: Labor migration dominates, with inconsistent employer-based health arrangements. One participant stated, *“Some employers pay for workers’ care*,* but many do not*,* leaving migrants with no safety net.”*

These differences shape both health needs and the feasibility of interventions: stable migration along the Lao PDR border allows more continuity of care, while highly mobile or undocumented groups at the Myanmar and Cambodia borders face persistent gaps.

### Theme 3: Health Priorities

Across all sites, respondents identified communicable diseases, maternal and child health, mental health, and occupational hazards as key concerns.

Myanmar border: Malaria, tuberculosis (TB), HIV/AIDS, and maternal complications were described as urgent issues. *“We still see high rates of malaria and TB in migrant populations*,* especially in the camps*,*”* said a district health officer.

Lao PDR border: Vaccination coverage was stronger, but gaps remained in child nutrition and maternal follow-up for seasonal migrants. *“When families move back and forth*,* it is difficult to keep track of children’s immunizations*,*”* noted a nurse.

Cambodia border: Occupational injuries in agriculture and construction were dominant, often without employer or insurance support. *“Many migrants come with injuries because they have no safety equipment and no insurance*,*”* explained an NGO representative.

Mental health concerns—stress, anxiety, and depression linked to insecurity and fear of arrest—were reported across all regions, though formal services remain scarce.

### Theme 4: Collaboration and Barriers

Migrant health volunteers (MHVs), village health volunteers (VHVs), and NGOs were widely recognized as essential for outreach, education, and referrals in hard-to-reach areas. MHVs are migrant who agreed to is a bridge between migrants and healthcare services. However, MHVs’ and NGOs’ roles remain poorly integrated into the health system, leading to duplication and inefficiency. A provincial officer at the Thai-Myanmar border reflected, *“We rely on MHVs and NGOs*,* but there is no clear mechanism to coordinate with government services.”*

Barriers to collaboration included legal restrictions, fear of arrest among undocumented migrants, and persistent language and cultural divides. These issues varied by border:

Myanmar border: NGOs are highly active in refugee camps, but collaboration with government facilities is minimal. Codes: “NGO-dependent,” “refugee camps,” “parallel systems.”

Lao PDR border: Cooperation is smoother due to stable migration and fewer legal conflicts. Codes: “seasonal migration cooperation,” “cross-border health programs.”

Cambodia border: Employer-mediated healthcare complicates coordination, as some employers fund care while others avoid responsibility. Codes: “employer decides care,” “no insurance support,” “fragmented employer role.”

Key informants agreed that formal platforms for coordination, shared data systems, and recognition of MHVs within the health system would significantly improve efficiency and sustainability of border health interventions.

### Theme 5: Feasibility of Engagement

Despite barriers, key informants at Myanmar border agreed that multi-stakeholder collaboration is both feasible and effective when coordinated. Mobile vaccination campaigns, cross-border surveillance, and NGO-supported maternal health programs were cited as successes. A district health officer from Myanmar border observed, *“Whenever we work with NGOs and migrant health volunteers*,* vaccination rates go up*,* and communities trust us more.”*

Yet, most initiatives remained donor-driven and temporary. As one NGO staff member in Myanmar border remarked, *“We can do a lot when we have government support*,* but the problem is that funding stops after the project ends.”* Participants stressed the need to institutionalize partnerships, secure sustainable financing, and formally integrate MHVs into official systems.

### Theme 6: Social Determinants and Risk Factors

Health access was shaped by intersecting social determinants:

Legal and administrative barriers excluded undocumented migrants from coverage. *“Undocumented migrants rarely come to health facilities because they fear being reported*,*”* explained a district officer at Myanmar border. Geographic isolation limited access for remote villages, especially in the mountainous border areas of Myanmar. Economic constraints pushed migrants toward informal care and self-medication. Cultural and language barriers reduced trust and hindered effective communication. Occupational risks expose Cambodian labor migrants to high injury rates without consistent employer support. Fragmented services from NGOs and MHVs remained donor-dependent. Together, these factors reinforced existing inequities and underscored the urgent need for inclusive policies, sustainable collaboration, and targeted interventions in border settings.

### Comparative Health System Dynamics Across Borders

We synthesized all thematic results and summarized them into two key differences in healthcare and factors influencing PHC systems in border areas (Table 2). The comparative analysis of Thailand’s borders with Myanmar, Lao PDR, and Cambodia (Table 2) reveals both everyday challenges and distinct dynamics that shape access to primary health care. Along the Myanmar border, migration is characterized by large numbers of undocumented migrants and long-standing refugee camps. Health services in these areas remain heavily reliant on NGOs, with minimal integration into government systems, resulting in fragmented and donor-dependent service delivery [3]. Geographic barriers—including mountainous terrain and poor transportation infrastructure—further restrict access. The main health issues identified were communicable diseases such as malaria, TB, and HIV/AIDS, alongside maternal and child health gaps in refugee camps. Legal exclusion, language barriers, and fear of arrest compound these vulnerabilities, positioning the Myanmar border as the most complex and resource-constrained setting.

**Table 2:** Key differences of health care and factors influencing PHC systems in border areas

Health Care in the Border Region			
Aspect	Thailand–Myanmar Border	Thailand–Lao PDR Border	Thailand–Cambodia Border
Migration Pattern	Large numbers of undocumented migrants and refugees in camps; irregular migration.	Seasonal and family-based migration, more stable mobility.	High labour migration, especially for agriculture and construction.
Accessibility	Many villages are remote, requiring extended travel to health facilities due to limited transportation infrastructure.	Easier land and river crossings; some remote areas, but better road access compared to the Myanmar border.	Relatively accessible by road, but border crossings are linked to trade and labor migration routes.
Health Service Access	NGOs heavily drive services among migrants and refugees; there is limited integration with government health facilities.	Better coordination between local authorities and NGOs in Thailand; more stable access to services.	Employer-mediated healthcare is common but inconsistent; many migrants remain uninsured.
Main Health Issues	High prevalence of malaria, TB, HIV/AIDS; maternal and child health gaps in camps.	Child malnutrition, gaps in maternal health follow-up due to mobility.	Occupational injuries, work-related illnesses, and communicable diseases in mobile labors.
Settlement Patterns	Presence of refugee camps and informal settlements near the border.	Rural farming communities with stable villages along the river.	Numerous agricultural settlements and labor camps for seasonal workers.
Cross-border Interaction	Frequent irregular crossings through informal routes due to porous borders.	Crossings often occur at formal checkpoints and are seasonal, linked to agriculture and family ties.	Cross-border trade and labor migration through both formal and informal crossings.
Collaboration Level	Fragmented, donor-dependent, with parallel systems between NGOs and government.	More structured bilateral agreements enabling joint programs.	Weak coordination; employers’ role in healthcare is inconsistent.
Key Barriers	Legal status issues, language barriers, rugged terrain, and fear of arrest.	Remote communities have less political sensitivity about migrants.	Lack of employer accountability, financial barriers, and fragmented services.

Factors Influencing PHC Systems in Border Areas			
Factor	Thailand–Myanmar Border	Thailand–Lao PDR Border	Thailand–Cambodia Border
Legal Status	Large numbers of undocumented migrants and refugees have limited health rights.	Fewer undocumented migrants than other borders; more seasonal and family-based migration.	Many undocumented labor migrants have employer-dependent access to care.
Geographic Barriers	Mountainous terrain, poor road networks, and remote settlements.	Riverine border with some remote villages, but better road access.	Mostly flat terrain, easier travel, but dispersed agricultural settlements.
Economic Constraints	Migrants with unstable incomes; heavy reliance on NGOs for free care.	Rural poverty and limited health resources in remote areas.	Migrant workers in agriculture/construction often lack insurance and rely on employers for healthcare.
Language & Culture	Diverse ethnic groups; language barriers hinder communication.	Ethnolinguistic diversity is present but less severe than on the Myanmar border.	Khmer-speaking migrants often face communication gaps with Thai providers.
Health Risks	High burden of malaria, TB, HIV/AIDS, and maternal health issues.	Gaps in maternal/child health follow-up and child malnutrition.	Occupational injuries, pesticide exposure, and communicable diseases among mobile labors.
Service Fragmentation	Heavy NGO involvement with limited integration into public health services.	Better cooperation across the countries through bilateral agreements.	Health access depends on inconsistent employer support and the availability of NGO services.

In contrast, the Lao PDR border is defined by seasonal, family-based migration and more stable settlement patterns in rural farming communities. While some villages are remote, road and river access is comparatively better than on the Myanmar border, enabling closer connections to health facilities [1]. Bilateral agreements between Thailand and Lao PDR have facilitated more coordinated health initiatives, particularly in maternal and child health. Nonetheless, child malnutrition and gaps in maternal follow-up care remain persistent issues. Unlike the Myanmar and Cambodia borders, political sensitivities are less pronounced, allowing for smoother cooperation between authorities and NGOs across the borders.

The Cambodia border presents a different picture, with high levels of labor migration into agriculture and construction. Migrants often live in dispersed labor camps and rely on employer-mediated health care, which remains inconsistent and poorly regulated. While road access is generally good, employer responsibility for health care is uneven, leaving many workers uninsured. Occupational injuries, pesticide exposure, and communicable diseases among mobile laborers dominate health risks in the Thailand-Cambodia area. Fragmented coordination, weak enforcement of labor protections, and financial barriers significantly limit access to care.

Taken together, these findings underscore that Thailand’s border regions share common challenges—such as service fragmentation, financial constraints, and cultural or linguistic barriers—but the relative importance of these factors differs across borders. The Myanmar border faces the most severe obstacles due to political instability and legal exclusion; the Lao PDR border benefits from more stable cooperation, and the Cambodia border is shaped by labor migration and employer-dependent health access. These differences highlight the necessity of context-specific strategies for strengthening PHC in border areas rather than a uniform national approach.

## Discussion

This study examined the structure, challenges, and opportunities of PHC systems in Thailand’s border regions with Myanmar, Lao PDR, and Cambodia. The findings reveal that border populations—particularly migrants, and refugees—face multiple, intersecting barriers to healthcare access, including legal exclusion, geographic isolation, economic insecurity, and cultural or linguistic divides. These challenges mirror global evidence demonstrating that migrant and displaced populations are disproportionately excluded from formal health systems due to structural and policy-level barriers [19–21].

### Interpretation of Findings

Border regions occupy a unique socio-political space where national health policies often excluded these vulnerable populations. Although Thailand’s Universal Health Coverage scheme has significantly expanded access and reduced out-of-pocket costs, its benefits do not consistently extend to undocumented migrants [19, 20]. Many participants described these populations as “falling through the cracks,” highlighting the tension between inclusive health policy goals and restrictive migration governance. This aligns with regional research showing that legal status is one of the strongest determinants of healthcare utilization in Southeast Asia [21].

Geographic isolation further compounds inequities. In remote mountainous districts—especially along the Myanmar border—limited transportation infrastructure and health workforce shortages delay care-seeking and outreach. Such conditions contribute to preventable morbidity and mortality, underscoring the critical role of mobile clinics, community-based health workers, and digital health innovations in improving service reach and timeliness [22, 23].

### Differences Across Borders

The study revealed marked contextual variations across Thailand’s three border zones.

The Myanmar border is characterized by long-standing refugee camps, large numbers of undocumented migrants, and dependence on NGO-led parallel systems. Here, health services remain fragmented and donor-driven, with minimal integration into government structures.

The Lao PDR border demonstrates greater stability through family-based migration and long-term bilateral cooperation, particularly in maternal and child health. The Cambodia border is shaped by labor migration, where employer-based or out-of-pocket payment models predominate, leading to inconsistent coverage and high occupational risk. These differences illustrate how migration typologies and governance frameworks shape the feasibility and sustainability of PHC interventions. They also reinforce the need for context-specific, locally adapted strategies, rather than uniform national approaches to border health governance [6, 24].

### Role of Multi-Sectoral Collaboration

Across all sites, respondents emphasized the indispensable role of migrant health volunteers, village health volunteers, and non-governmental organizations in extending health services to marginalized populations. These actors often serve as cultural mediators and trusted community links, facilitating communication, referral, and disease prevention efforts. This finding aligns with global literature highlighting the contribution of community-based health workers in improving health outcomes among underserved populations [25].

However, collaboration remains fragmented and donor-dependent. Without formal mechanisms for coordination, MHVs and NGOs frequently operate in parallel to government systems, leading to duplication in some areas and service gaps in others. Such fragmentation reflects broader governance challenges in cross-border health management, where institutional responsibilities are dispersed and funding is short-term [26].

Sustainable collaboration requires formalized frameworks that integrate non-state actors into Thailand’s PHC system, define roles and accountability structures, and ensure continuity beyond project cycles. Experiences from provinces such as Tak and Ranong demonstrate that coordinated partnerships can enhance vaccination coverage, maternal health outcomes, and community trust when aligned with local administrative support.

### Feasibility of Multi-Stakeholder Engagement

Findings suggest that multi-stakeholder engagement in border health governance is both feasible and beneficial when grounded in mutual trust, shared objectives, and policy alignment. Inclusive engagement enhances cultural sensitivity, responsiveness, and community ownership—key elements of people-centered care. Successful models in Tak, Ranong, Trat, and Sa Kaeo provinces demonstrate that collaboration between local authorities, NGOs, and civil society can improve access for migrant populations.

Nevertheless, such partnerships often lack institutional backing and are vulnerable to funding discontinuities. Structural barriers—such as unclear mandates, linguistic divides, and unequal power relations—limit sustained coordination. Establishing formal multi-sectoral platforms at provincial and district levels, embedded within national UHC and border health policies, would provide an enabling environment for continuous collaboration and joint decision-making [27].

### Social Determinants of Health and Equity Implications

Health access in border regions is deeply influenced by social determinants that extend beyond the health sector. Legal and administrative barriers exclude undocumented migrants from insurance schemes, reinforcing fear of deportation and deterring timely care [28]. Physical isolation in mountainous and rural areas further restricts mobility and outreach, particularly along the Myanmar frontier [29]. Economic insecurity forces migrants to rely on informal providers or delay treatment, while employer-provided health support—especially along the Cambodian border—remains inconsistent and weakly regulated.

Cultural and linguistic barriers also undermine provider-patient communication and trust, reducing adherence to treatment [30]. These determinants interact to produce structural inequities that conventional, facility-based health systems struggle to address. Greater integration of MHVs through formal recognition, standardized training, and financial compensation could enhance both cultural competence and sustainability in all border areas [31, 32].

Broader governance and policy dynamics, including shifting migration laws and fragmented donor priorities, also influence system responsiveness. Addressing these determinants requires whole-of-government and whole-of-society approaches that link health, labor, and migration policies under a shared equity agenda.

### Study Strengths and Limitations

This study’s strengths include its multi-site design across Thailand borders and three neighboring countries and its inclusion of diverse stakeholder perspectives—health providers, administrators, NGOs, and community representatives—offering a comprehensive view of cross-border PHC dynamics. Triangulation and peer debriefing enhanced the credibility of findings.

However, several limitations should be acknowledged. The use of purposive and snowball sampling may have introduced selection bias, potentially overrepresenting certain institutional perspectives. Reliance on secondary data and qualitative self-reporting limited the capacity to assess intervention outcomes quantitatively. Nonetheless, the findings provide valuable insight into the institutional, social, and policy determinants that shape PHC for border populations and identify key leverage points for reform.

## Conclusion

This study highlights the complex interplay of structural, social, and governance factors shaping primary health care in Thailand’s border regions with Myanmar, Lao PDR, and Cambodia. While Thailand’s universal health coverage framework has achieved impressive national gains, the border context reveals persistent inequities rooted in migration status, geographic isolation, and fragmented coordination. Strengthening PHC in these regions therefore requires strategies that are inclusive, adaptive, and grounded in cross-sectoral collaboration.

Integrating migrant and community health volunteers into formal PHC structures, institutionalizing multi-stakeholder coordination platforms, and ensuring sustainable financing mechanisms are key steps toward closing existing coverage gaps. Tailored, border-specific approaches—aligned with local migration patterns and social realities—will be essential to ensure that the most vulnerable populations are not left behind.

Beyond Thailand, these findings hold broader relevance for other borderland and mobile population settings worldwide. Similar challenges exist in other Greater Mekong Subregion (GMS) areas, such as the Lao PDR–Vietnam and Cambodia–Vietnam borders, where informal migration and donor-dependent services mirror the Thai experience.

By applying a people-centered health systems (PCHS) lens to understand border health, policymakers and practitioners can design PHC strategies that extend beyond national boundaries—linking health equity with migration management, social protection, and regional cooperation. Ultimately, achieving universal health coverage in border settings demands not only technical solutions but also political commitment and institutional innovation to bridge the divides between health systems, communities, and nations.

## Data Availability

No datasets were generated or analysed during the current study.
